# Spontaneous Emergence of Azithromycin Resistance in Independent Lineages of *Salmonella* Typhi in Northern India

**DOI:** 10.1093/cid/ciaa1773

**Published:** 2021-01-30

**Authors:** Megan E Carey, Ruby Jain, Mohammad Yousuf, Mailis Maes, Zoe A Dyson, Trang Nguyen Hoang Thu, To Nguyen Thi Nguyen, Thanh Ho Ngoc Dan, Quynh Nhu Pham Nguyen, Jaspreet Mahindroo, Duy Thanh Pham, Kawaljeet Singh Sandha, Stephen Baker, Neelam Taneja

**Affiliations:** 1 Cambridge Institute of Therapeutic Immunology and Infectious Disease (CITIID), Department of Medicine, University of Cambridge, Cambridge, United Kingdom; 2 Civil Hospital, Manimajra, Chandigarh, India; 3 Department of Medical Microbiology, Postgraduate Institute of Medical Education and Research, Chandigarh, India; 4 Department of Infectious Diseases, Central Clinical School, Monash University, Melbourne, Victoria, Australia; 5 London School of Hygiene and Tropical Medicine, London, United Kingdom; 6 The Hospital for Tropical Diseases, Wellcome Trust Major Overseas Programme, Oxford University Clinical Research Unit, Ho Chi Minh City, Vietnam; 7 Centre for Tropical Medicine and Global Health, University of Oxford, Oxford, United Kingdom

**Keywords:** *Salmonella* Typhi, typhoid fever, antimicrobial resistance, azithromycin resistance, India

## Abstract

**Background:**

The emergence and spread of antimicrobial resistance (AMR) pose a major threat to the effective treatment and control of typhoid fever. The ongoing outbreak of extensively drug-resistant *Salmonella* Typhi (*S*. Typhi) in Pakistan has left azithromycin as the only remaining broadly efficacious oral antimicrobial for typhoid in South Asia. Ominously, azithromycin-resistant *S*. Typhi organisms have been subsequently reported in Bangladesh, Pakistan, and Nepal.

**Methods:**

Here, we aimed to understand the molecular basis of AMR in 66 *S*. Typhi organisms isolated in a cross-sectional study performed in a suburb of Chandigarh in Northern India using whole-genome sequencing and phylogenetic analysis.

**Results:**

We identified 7 *S*. Typhi organisms with the R717Q mutation in the *acrB* gene that was recently found to confer resistance to azithromycin in Bangladesh. Six out of the seven azithromycin-resistant *S*. Typhi isolates also exhibited triple mutations in *gyrA* (S83F and D87N) and *parC* (S80I) genes and were resistant to ciprofloxacin. These contemporary ciprofloxacin/azithromycin-resistant isolates were phylogenetically distinct from each other and from those reported from Bangladesh, Pakistan, and Nepal.

**Conclusions:**

The independent emergence of azithromycin-resistant typhoid in Northern India reflects an emerging broader problem across South Asia and illustrates the urgent need for the introduction of typhoid conjugate vaccines in the region.

## Teaser

We identified ciprofloxacin/azithromycin-resistant *Salmonella* Typhi in Chandigarh in Northern India. The independent emergence of ciprofloxacin/azithromycin-resistant typhoid in Bangladesh, Pakistan, Nepal, and India and the continued spread of extensively drug-resistant typhoid in Pakistan highlight the limitations of licensed oral treatments for typhoid fever in South Asia.

*Salmonella enterica* serovar Typhi (*S*. Typhi), the etiologic agent of typhoid fever, is associated with an estimated 10.9 million infections and 116 800 deaths globally [1]. The majority of this disease burden is concentrated in South Asia, which has a modeled incidence rate of 592 cases per 100 000 person-years [1]. A pooled estimate of typhoid fever incidence of 377 cases per 100 000 in India has also been calculated using limited population-based data; significant geographical heterogeneity was observed [2]. The ongoing Surveillance for Enteric Fever in India (SEFI) Study is generating geographically representative, age-specific incidence data, as well as additional information regarding cost of illness, range of clinical severity, and antimicrobial resistance (AMR) patterns associated with *S*. Typhi in India [3]. These data will undoubtedly provide a more comprehensive understanding of typhoid fever incidence rates across the Indian subcontinent and, ultimately, in supporting decision making concerning typhoid conjugate vaccine (TCV) introduction in India [4].

Growing rates of AMR have made typhoid control increasingly challenging, beginning with the rise of multidrug resistance (MDR; resistant to chloramphenicol, trimethoprim-sulfamethoxazole, ampicillin) in the 1990s [5] and the subsequent increase in fluoroquinolone resistance in the early 2000s, which was predominantly focused in South and Southeast Asia [6, 7]. Ultimately, these phenotypic changes led to the common use of third-generation cephalosporins for the treatment of typhoid fever. The emergence and spread of extensively drug-resistant (XDR; resistant to chloramphenicol, ampicillin, cotrimoxazole, streptomycin, fluoroquinolones, and third-generation cephalosporins) typhoid in Pakistan has left azithromycin as the only available oral antimicrobial for effective treatment of typhoid fever across South Asia [8]. Concerningly, azithromycin-resistant *S*. Typhi have subsequently been reported in Bangladesh, Pakistan, and Nepal, although this phenotype has not yet arisen in XDR organisms [9–11].

It is apparent that we have an escalating problem with drug-resistant *S*. Typhi in South Asia, due in part to empirical treatment of febrile patients and widespread community availability of antimicrobials. We are currently unsure of the regional distribution of azithromycin resistance or the potential for the emergence of a specific sublineage with this phenotype, as has been observed for MDR, XDR, and fluoroquinolone resistance. Here, we aimed to characterize the molecular basis of AMR in *S*. Typhi in a cross-sectional study performed in a suburb of Chandigarh in Northern India. Through whole-genome sequencing (WGS), we describe the distribution of a collection of azithromycin-resistant *S*. Typhi and show that these organisms have arisen independently of those in Pakistan and Bangladesh through the acquisition of an identical mutation in the *arcB* gene. Our data support the prioritization of TCV introduction to prevent the continued emergence and spread of drug-resistant *S*. Typhi in South Asia.

## METHODS

### Ethics

Ethical clearance was granted by the Institutional Ethics Committee of the Government Multi-specialty Hospital, Sector 16, Chandigarh (letter no. GMSH/2018/8763 dated 26 July 2018) and the Postgraduate Institute of Medical Education and Research (PGIMER) Institutional Ethics Committee (IEC-08/2018–285 dated 24 September 2018). Administrative approval to carry out this collaborative study on enteric fever was granted by the Chandigarh Health Department (CHMM-2017/2991 dated 28 August 2017). Approval was also granted by the Collaborative Research Committee of PGIMER (no. 79/227-Edu-18/4997 dated 12 December 2018). Informed consent was a prerequisite for inclusion in the study.

### Study Design

The *S*. Typhi isolates were obtained from blood cultures taken from febrile patients presenting to Civil Hospital Manimajra in Chandigarh (CHMM) between September 2016 and December 2017, where passive blood culture surveillance has been conducted since November 2013. The CHMM is a 100-bed secondary healthcare facility located on the outskirts of Chandigarh, and serves a catchment area of approximately 200 000 people, including referrals from 4 primary care centers. Data from patients with blood culture–confirmed invasive *Salmonella* infection, which includes *Salmonella* serovars Typhi and Paratyphi A, B, and C, from both inpatient and outpatient wards were used in the study. Clinical history, laboratory test results, and risk factor data were recorded for each confirmed case of enteric fever for patients residing in Manimajra. This analysis focuses solely on confirmed cases of *S*. Typhi.

### Identification and Antimicrobial Susceptibility Testing

Bacterial isolates were identified as *S*. Typhi using conventional biochemical tests; motility agar, Hugh–Leifson Oxidative-Fermentation test, the Triple Sugar Iron test, citrate test, urease test, phenyl pyruvic acid test, and indole test. All isolates were eventually confirmed using antisera from Central Research Institute, Kasauli. Antimicrobial susceptibility was determined for the following antimicrobials by disc diffusion: ampicillin (10 μg), chloramphenicol (30 μg), trimethoprim/sulfamethoxazole (1.25/23.75 μg), ceftriaxone (30 μg), azithromycin (15 μg), ciprofloxacin (5 μg), and pefloxacin (5 μg). Zone diameters were measured and interpreted as per Clinical Laboratory Standards Institute guidelines [12]. Minimum inhibitory concentration (MIC) testing was also conducted on all organisms showing resistance to any of the above antimicrobials by disc diffusion using E-tests (bioMerieux, France).

### Whole-Genome Sequencing and Phylogenetic Analysis

All *S*. Typhi were stored and shipped to PGIMER Chandigarh. Isolates from patients residing outside of Manimajra were excluded, as were those for which there were inadequate clinical metadata, or the DNA yield was below the amount required for WGS. Total genomic DNA was extracted from the *S.* Typhi using the Wizard genomic DNA extraction Kit (Promega, Madison WI, USA) and subjected to WGS using the Illumina MiSeq platform (Illumina, San Diego, CA, USA) to generate 250-bp paired end reads. We then aimed to put these sequences into global and regional phylogenetic context. Subsequently, these reads were mapped against the CT18 reference sequence (accession no. AL513382) using the RedDog mapping pipeline (V1beta.10.4; available at: https://github.com/katholt/RedDog) to identify single nucleotide variants (SNVs) [13–15]. RedDog uses Bowtie (v2.2.9) [16] to map reads to the reference sequence; SAMtools (v1.9) [17] is used to identify SNVs with phred quality scores above 30; to filter out SNVs supported by less than 5 reads, or with more than 2.5 times the genome-wide average read depth (representing putative repeated sequences), or with ambiguous (heterozygous) consensus base calls. For each SNV position that passed these criteria in any single sequence, consensus base calls/alleles for that position were extracted from all genomes and used to construct an alignment of alleles across all SNV sites. Ambiguous base calls and those with a phred quality score of less than 20 were treated as unknown alleles and represented with a gap character in the SNV alignment. Read alignments were used to assign isolates to previously defined lineages according to the extended genotyping framework [13] by subjecting the alignments Binary Alignment Map (BAM format) to analysis using the GenoTyphi pipeline (available at: http://github.com/katholt/genotyphi).

Chromosomal SNVs with confident homozygous calls (phred score >20) in more than 95% of the genomes mapped (representing a “soft” core genome) were concatenated to form an alignment of alleles at 26 991 variant sites, and alleles from *S*. Paratyphi A str. AKU1_12601 (accession no. FM200053) [18] were also included using the same mapping approach for phylogenetic tree outgroup rooting. SNVs called in repetitive sequences and prophage regions were removed from the alignment using the parseSNPtable.py script from RedDog (354 kbp; ~7.4% of bases in the CT18 reference chromosome, as defined previously) [13, 19, 20] and a pseudo-genome alignment inferred using the CT18 reference sequence with the snpTable2GenomeAlignment.py script (also from RedDog). Any further recombination was filtered from the resultant whole-genome pseudo-alignment using Gubbins (v2.3.2) [21], resulting in a final alignment length of 25 832 chromosomal SNVs for 3473 isolates.

RAxML (v8.9.2) [22] was used to infer maximum likelihood (ML) phylogenetic trees from the final chromosomal SNV alignment, with a generalized time-reversible model, a gamma distribution to model site-specific rate variation (the GTR+ Γ substitution model; GTRGAMMA in RAxML), and 100 bootstrap pseudo-replicates to assess branch support. Resultant ML trees were visualized with Microreact [23] (https://microreact.org/project/nniNzBL2uq3XZXYDKgG374) and the Interactive Tree of Life [24]. SRST2 (v0.2.0) [25] was used with ARGannot (available at: https://github.com/katholt/srst2/blob/master/data/ARGannot_r3.fasta) [26] and PlasmidFinder (available at: https://github.com/katholt/srst2/blob/master/data/PlasmidFinder.fasta) [27] databases to identify AMR genes and plasmid replicons, respectively. Mutations in *gyrA* and *parC*, as well as the R717Q mutation in *acrB*, were detected using GenoTyphi (https://github.com/katholt/genotyphi). Raw read data were deposited in the European Nucleotide Archive (ENA)under accession number ERP124488.

## RESULTS

### Epidemiological Observations

**Figure 1.**
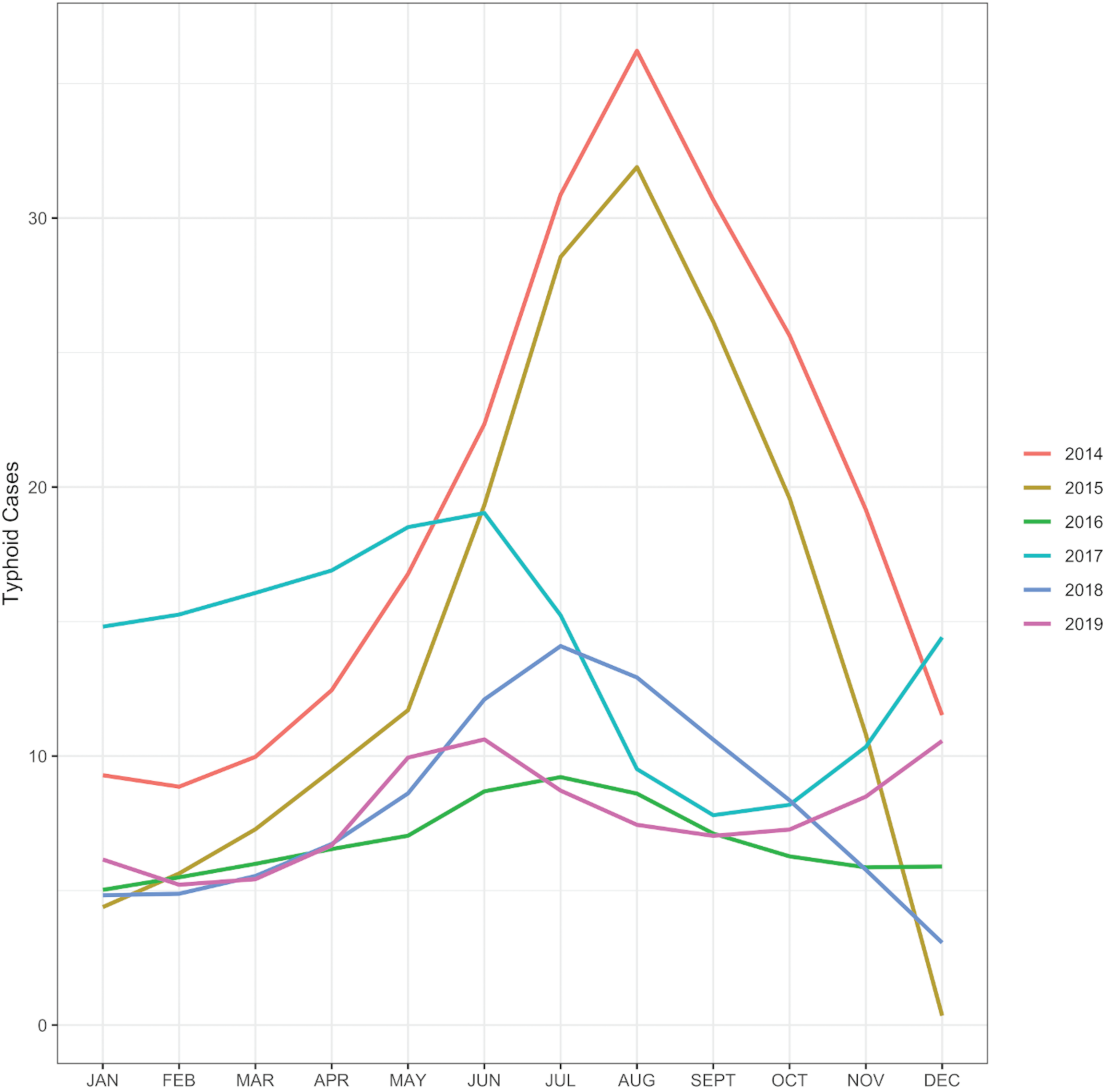
The annual seasonality of typhoid fever in Manimajra, Chandigarh. Plots showing the number of typhoid cases recorded the civil hospital in Manimajra from 2014 to 2019. The observed annual peak in typhoid cases corresponds with the monsoon season in Northern India (May to September).

Typhoid fever is a major public health concern among children and young adults in this region of Northern India. It is thought that many cases in this area, a hub for the states of Punjab, Haryana, and Himachal Pradesh, are associated with the mixing of large populations of seasonal workers from the adjoining states. These workers generally live in informal dwellings with poor sanitation and limited access to safe water. Approximately 1500 patients with suspected enteric fever present to the CHMM facility annually and receive blood cultures, of which approximately 10% are positive for *S*. Typhi. There is a seasonal peak of typhoid fever in this facility during the monsoon months from May to September (Figure 1). As a component of a typhoid surveillance program at CHMM, study staff provided health education about safe drinking water and chlorine tablets to all households where there was a confirmed typhoid fever case from mid-2017 onwards. This likely accounts for the reduction in case numbers from 2017 to 2019.

**Figure 2.**
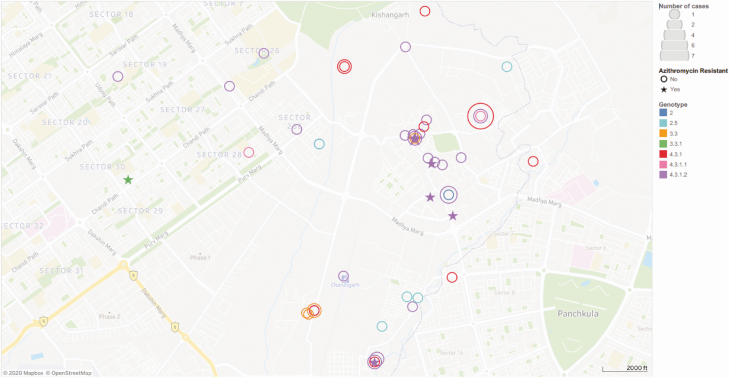
The spatial distribution of confirmed typhoid fever cases in Chandigarh. Map of Chandigarh (scale shown) of the residential locations of the *Salmonella* Typhi cases recorded at the civil hospital in Manimajra between September 2016 and December 2017. *S*. Typhi genotype is indicated by color (see key), azithromycin-resistant isolates are indicated with stars, and the number of cases in each coordinate is represented by the size of the circles. All cases were located within a 28-km^2^ area. There is a cluster of cases of genotype 4.3.1.2 in a 0.25-km^2^ area of central Manimajra, which includes 5 of the 6 closely related azithromycin-resistant isolates.

The *S*. Typhi organisms interrogated here by WGS were isolated between September 2016 and December 2017 and all originated from blood cultures taken from febrile patients attending CHMM. All patients with a positive blood culture for *S*. Typhi resided within 12.9 km of the healthcare facility and were located in an area of approximately 28 km^2^ (Figure 2). *S.* Typhi was isolated throughout the specified months, again with a higher number of cases observed between May and September. The median age of patients with typhoid included in this analysis was 7 years. The standard-of-care antimicrobials at this facility for patients with suspected enteric fever in outpatient settings are cotrimoxazole, cefixime, and/or azithromycin, and ceftriaxone for inpatients.

### The Local Phylogenetic Structure of *S.* Typhi

**Figure 3.**
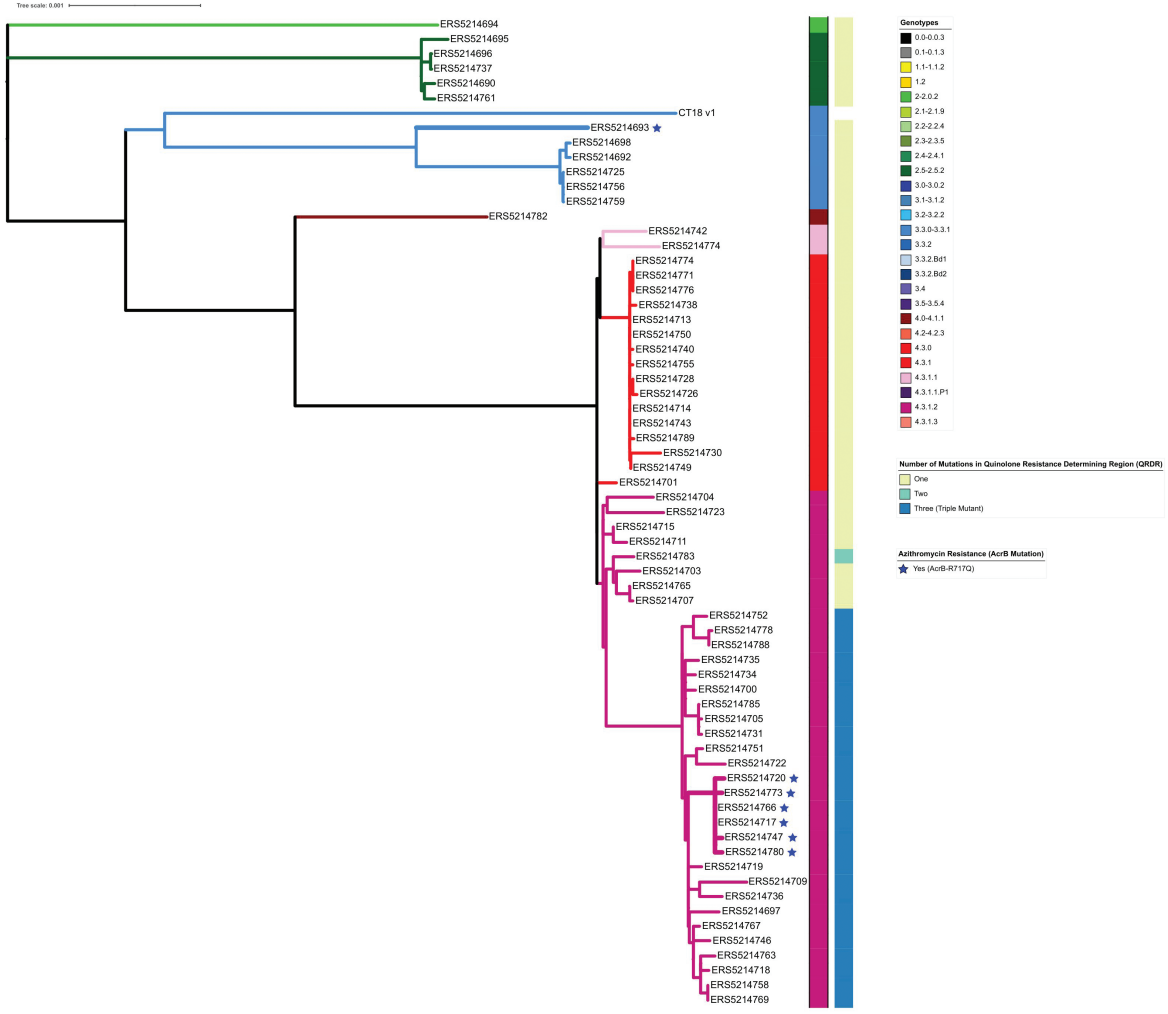
The phylogenetic distribution of *Salmonella* Typhi isolated at the civil hospital in Manimajra, Chandigarh. Phylogenetic tree made in RAxML of the 67 isolates genome sequenced. This collection shows considerable genetic diversity, with 8 genotypes represented (as color coded on branches and in the key). Mutations in the Quinolone Resistance Determining Region (QRDR) and presence of the acrB-R717Q mutation are shown for each organism. There are 2 distinct clusters of organisms with the *acrB* mutation that confers azithromycin resistance; each of these individual organisms are indicated with a star.

Ultimately, after data quality control, we generated and analyzed 66 *S*. Typhi genome sequences from Chandigarh. We observed that the population structure of *S*. Typhi around Chandigarh exhibited a high level of genetic diversity with 8 co-circulating genotypes, indicative of population mixing and sustained introduction of organisms from a variety of locations across India (Figure 3). However, and in an analogous manner to other locations in Asia (and East Africa), most organisms (80%, 53/66) belonged to lineage 4.3.1 (H58), with the majority of those (66%, 35/53) belonging to 4.3.1.2. In total, 24% (16/66) of isolates were subclade 4.3.1 and 3% (2/66) were 4.3.1.1. Additional genotypes were composed of subclade 3.3 (7.5%, 5/66), clade 2.5 (7.5%, 5/66), clade 3.3.1 (1.5%, 1/66), clade 4.1 (1.5%, 1/66), and major lineage 2 (genotype 2; 1.5%, 1/66).

### Fluoroquinolone Resistance

All (66/66) *S*. Typhi genome sequences, regardless of the genotype, possessed mutations in *gyrA*, conferring reduced susceptibility to fluoroquinolones. Notably, given that these mutations were observed in a range of genotypes, these had occurred independently, likely as a result of sustained antimicrobial pressure from widespread fluoroquinolone use. We further observed multiple *gyrA* mutation profiles in 4.3.1 organisms conferring intermediate resistance against fluoroquinolones (0.12 μg/mL < ciprofloxacin MIC < 1 μg/mL). These mutations included S83Y (29.1%, 16/55), S83F (16.4%, 9/55), and D87N (1.8%, 1/55). Additionally, we identified a subclade of organisms that represented 49.1% (27/55) of the 4.3.1 isolates, all of which belonged to 4.3.1.2, that contained the classical triple mutations associated with fluoroquinolone resistance (S83F and D87N in *gyrA* and S80I in *parC*) [14]. These organisms exhibited high-level fluoroquinolone resistance (ciprofloxacin MIC >24 μg/mL). Our observations with respect to ubiquitous fluoroquinolone resistance were concerning; however, none of the *S*. Typhi isolates were MDR, which may be associated with a reduced reliance on older classes of antimicrobials.

### Azithromycin Resistance

We identified that 7 of 66 (10.6%) of the sequenced isolates contained a mutation in *acrB*, a gene encoding a component of the AcrAB efflux pump [28]. Mutations in *acrB* have been previously observed to be associated with resistance to azithromycin [9]. There are currently no other described mechanisms of azithromycin resistance in *S*. Typhi. Here, the *acrB* mutation was nonsynonymous (R717Q) and identified in 6 genotype 4.3.1.2 organisms and in 1 genotype 3.3.1 organism. These data are indicative of convergent mutation in different lineages, highlighting a potential increasing reliance on azithromycin; this selective pressure is further accentuated by the small clonal expansion in genotype 4.3.1.2 (Figure 3).

**Figure 4.**
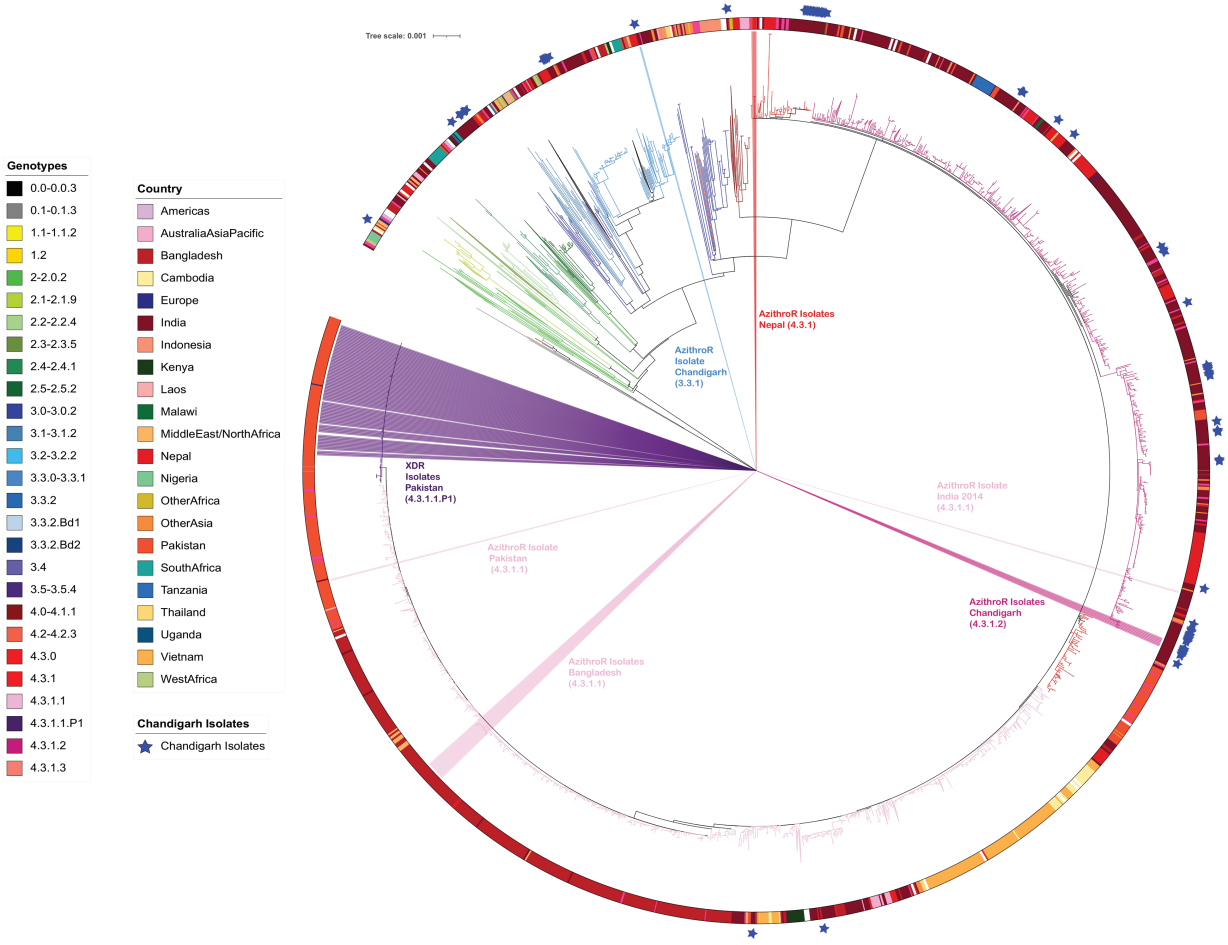
Azithromycin-resistant *Salmonella* Typhi in a global context. The diagram depicts a maximum likelihood rooted phylogenetic tree with a final alignment of 25 832 chromosomal SNVs for 3472 globally representative isolates, including all publicly available isolates from India. The color of the internal branches represents the genotype, the colored ring around the tree indicates the country or region of origin for each isolate, and the blue stars indicate which isolates originate from this study. Additionally, the tree contains each known *S*. Typhi isolate with an *acrB* mutation in public databases, these originate from India, Nepal, Bangladesh, and Pakistan. The location of the XDR isolates from Pakistan are added for context. Abbreviations: AzithroR, azithromycin resistant; SNV, single nucleotide variant; XDR, extensively drug-resistant.

The R717Q mutation in *acrB* has been linked to high azithromycin MICs in genotype 4.3.1.1 *S*. Typhi isolates from Bangladesh [8] and also in genotype 4.3.1.1 *S*. Typhi in Pakistan [9]. Here, 6 of 7 of the contemporary Indian *S*. Typhi isolates identified here with the same R717Q mutation in *acrB* showed nonsusceptibility to azithromycin with MICs greater than 16 μg/mL (Table 1). Our data suggested that these R7171Q mutations in the *acrB* gene have arisen spontaneously in India. To test this hypothesis, we constructed an expanded phylogenetic tree comprising a global *S.* Typhi collection, including organisms from across South Asia and the recently described azithromycin-resistant organisms from Bangladesh, Pakistan, and Nepal. We found that the azithromycin-resistant *S*. Typhi from India were phylogenetically distinct from those reported from Bangladesh, Pakistan, and Nepal (Figure 4). Additionally, we found that the azithromycin-resistant organisms associated with *acrB* mutations were dispersed around the tree and appear to have arisen on at least 6 different occasions, with a differing *acrB* mutation in organisms from Nepal (R717L) [11].

**Table 1.** *Salmonella* Typhi Considered Nonsusceptible to Azithromycin Identified in This Study

ENA Accession Number	Study ID^a^	MIC,^b^ μg/mL
ERS5214693	B950_2017	>256
ERS5214717	B628_2017	24
ERS5214720	B550_2017	>256
ERS5214747	B396_2017	64
ERS5214766	B231_2017	16^c^
ERS5214773	B117_2017	>256
ERS5214778	B113_2017	128

Abbreviations: ENA, European Nucleotide Archive; MIC, minimum inhibitory concentration.

^a^All organisms contained *acrB* mutation.

^b^Clinical and Laboratory Standards Institute (CLSI) guidelines for azithromycin susceptibility (MIC): susceptible, ≤16 µg/mL; resistant, ≥32 µg/mL.

^c^Contained *acrB* mutation but borderline susceptible by MIC.

The azithromycin-resistant isolates from India described here were isolated in 2017, meaning that they are contemporaneous with those reported from Bangladesh and Pakistan, and arose independently in phylogenetically distinct lineages. Last, 6 of the 7 Indian isolates with the R717Q mutation in *arcB* (all 4.3.1.2) were also within the group of organisms with the triple mutation associated with high-level fluoroquinolone resistance, making these organisms highly resistant to these 2 key oral antimicrobials.

## DISCUSSION

In this study, we aimed to describe the genomic aspects the *S*. Typhi causing disease in an endemic region in Northern India. We investigated antimicrobial susceptibility patterns using phenotypic testing and WGS data and then placed these data into a regional and global context using published genomic data. Notably, we identified 7 azithromycin-resistant *S*. Typhi isolates. These organisms belonged to 2 different lineages and were genetically distinct from azithromycin-resistant isolates recently reported from Bangladesh, Pakistan, and Nepal. Our observations suggest that azithromycin-resistance mutations at codon 717 in *arcB* are arising independently in locations where there is substantial selective pressure induced by azithromycin. At present, azithromycin resistance in *S*. Typhi is rare, as the 24 *acrB* mutants concatenated here represent all published *S*. Typhi sequences with this mutation. An increased reliance on azithromycin for treatment of typhoid fever (given in a 500-mg dose twice a day on day 1 and once a day for 2 weeks for adults and as a 10-mg/kg dose once a day for 2 weeks in children in this location) and other invasive bacterial infections in South Asia appears to be driving resistance. We also speculate that this may be facilitated by an interaction with fluoroquinolone-resistance mutations, which may stimulate the acquisition of further resistance phenotypes [29]. Additionally, ongoing clinical trials measuring the impact of prophylactic administration of azithromycin on growth and mortality of infants and young children in Pakistan, Bangladesh, and India [30] signal the inevitability of what Hooda and colleagues [30] have termed pan-oral drug-resistant Typhi. This scenario would necessitate inpatient intravenous drug administration for effective treatment of typhoid fever in the region at enormous cost to patients and to healthcare systems. Where intravenous drug administration is not an option, typhoid could once again become a disease with a high mortality rate, as was observed in the pre-antimicrobial era.

While the catchment area of this study is not representative of the entire Indian subcontinent, the phenomenon described herein is unlikely to be restricted to Chandigarh. India is currently the largest consumer of antimicrobials of all low- and middle-income countries, with a reported 6.5 billion defined daily doses (DDDs) in 2015, or 13.6 DDDs per 1000 inhabitants per day [31, 32]. With such widespread availability and use of antimicrobials nationally, selective pressure on circulating pathogens is likely immense. The SEFI study will soon yield additional concrete AMR data from multiple, geographically representative sites in India, which will further elucidate AMR patterns across the country. Ideally, these data will inform local antimicrobial stewardship practices and may be used as a basis for prioritization of future interventions.

How then to tackle this “red queen” dilemma? Drug-development efforts cannot keep pace with bacterial evolution. Therefore, there is an urgent need for preventative interventions, namely water, sanitation, and hygiene (WASH) interventions and TCV introduction, in India and across South Asia. There is also a need for enhanced typhoid surveillance, in South Asia and globally, specifically to monitor the emergence and spread of this and other resistance phenotypes. Historic genomic data show us that drug-resistant *S*. Typhi lineages emerge in South Asia and then spread to East Africa and even Latin America [33]. With the widespread prophylactic deployment of azithromycin through clinical studies and public health programs in West Africa and South Asia [30, 34] it will be critical to monitor global AMR patterns to mitigate a public health catastrophe. Recently published data from the MORDOR (Macrolides Oraux pour Réduire les Décès avec un Oeil sur la Résistance) cluster-randomized trial in Niger showed that a random sample of children (ages 1–59 months) living in villages randomized to receive a single dose of azithromycin twice annually through mass drug administration were 7.5 times more likely (95% confidence interval, 3.8–23.1) to have macrolide-resistance determinants in their gut microbiomes after 48 months than their peers residing in villages that were randomized to receive a placebo [35]. These data further underscore the need for continued and intensified AMR surveillance in these and other areas where community azithromycin use is high.

This emerging problem additionally represents an opportunity for the use of genomics to inform policy. Whole-genome sequencing data provide clear information regarding AMR in organisms where molecular mechanisms of resistance are understood. Genomic surveillance also enables the identification and characterization of new resistance phenotypes, as was the case for XDR typhoid [8] and the new azithromycin-resistant organisms identified in Bangladesh and described further here [9]. The outputs of antimicrobial susceptibility testing are not always straightforward, particularly in cases where susceptibility breakpoints have not been validated extensively using clinical data, as is the case for azithromycin [36]. Genomic AMR data can inform prioritization of TCV introduction, as well as implementation of WASH interventions. Genomic surveillance should also be an important component of long-term monitoring of the impact of widespread TCV deployment. Not only can genomic surveillance provide additional information on the impact of TCV on AMR it will also illustrate the impact of vaccine on bacterial population structures and enable the identification of any vaccine escape mutants. Such information is vital to understanding the long-term impact of vaccine and to facilitate any potential future efforts for global typhoid elimination.

## Notes

***Acknowledgments.*** The authors thank the patients in Manimajra who participated in the study and the healthcare workers who screened and enrolled patients.

***Disclaimer.*** The funders had no role in the design and conduct of the study; collection, management, analysis, and interpretation of the data; preparation, review, or approval of the manuscript; or decision to submit the manuscript for publication.

***Financial support.*** This work was supported by a Wellcome Trust Senior Research Fellowship to S. B. to (215515/Z/19/Z). D. T. P. is funded as a leadership fellow through the Oak Foundation. M. M. is funded by the National Institute for Health Research [Cambridge Biomedical Research Centre at the Cambridge University Hospitals NHS Foundation Trust]. The views expressed are those of the authors and not necessarily those of the NHS, the NIHR or the Department of Health and Social Care.

***Potential conflicts of interest.*** The authors: No reported conflicts of interest. All authors have submitted the ICMJE Form for Disclosure of Potential Conflicts of Interest.
